# Is vector control needed to eliminate gambiense human African trypanosomiasis?

**DOI:** 10.3389/fcimb.2013.00033

**Published:** 2013-07-31

**Authors:** Philippe Solano, Steve J. Torr, Mike J. Lehane

**Affiliations:** ^1^Institut de Recherche pour le Développement, UMR 177 IRD-CIRAD INTERTRYP, CIRDESBobo-Dioulasso, Burkina Faso; ^2^Vector Biology Department, Liverpool School of Tropical MedicineLiverpool, UK; ^3^Warwick Medical School, University of WarwickCoventry, UK

## Introduction

Human African Trypanosomiasis (HAT), or sleeping sickness, is a neglected tropical parasitic disease of humans due to trypanosomes transmitted by tsetse flies (*Glossina spp*.) in sub-Saharan Africa. Comparable diseases (Animal African Trypanosomiasis—AAT—nagana) are present in domesticated animals and these are an important constraint to animal health and production in Africa (Jordan, 1986; Kabayo, 2002). For HAT, there is no vaccine, no chemoprophylaxis, and treatment is still long and difficult to administer despite recent improvements (Simarro et al., 2012). In most cases HAT is fatal if untreated. The disease affects rural communities in remote parts of Africa, particularly people working outdoors (e.g., farmers, foresters, fishermen, people collecting water) and hence at greater risk of being bitten by tsetse. Two flagellate protozoan parasites cause HAT. *Trypanosoma brucei rhodesiense* causes the *rhodesiense* form of the disease (currently <5% of all cases) in eastern and southern Africa, and *T. b. gambiense* causes the *gambiense* form of the disease (currently >95% of all cases) in Central and West Africa (Simarro et al., 2010).

Although it is accepted that tsetse control plays a central role in combatting the *rhodesiense* form of HAT (Welburn et al., 2009), this has not been the case for the *gambiense* form. Indeed in the strategy recommended by WHO to control sleeping sickness, active case detection and treatment has always been the first, it not the only method recommended, until very recently. Historically, there have been two clear justifications for this—(1) that vector control is not required and/or that (2) it is too expensive and difficult to organize; we will discuss both.

## Is vector control required in gambiense hat control?

First, we would like to make it clear that we recognize that active case detection and treatment has proved effective in HAT control in many foci and that it is a necessary intervention if those infected are not to die. However, there are several foci where active case detection and treatment without vector control, despite saving many lives, has failed to bring HAT under effective control. Artzrouni and Gouteux (1996) developed a mathematical model of the basic Reproductive Number (*R*_0_) to analyse and compare vector control and active case detection and treatment in the control of *gambiense* HAT (see also Gouteux and Artzrouni, 1996). They showed that when transmission rates are high (strongly influenced by fly biting rates on humans) vector control is a requirement. The model was able to predict the failure of case detection and treatment and the need for the addition of vector control before control was achieved in the well-studied HAT focus of Niari, Congo Brazzaville. It is quite possible that the problem in other HAT foci which have proven intractable by active case detection and treatment may be explained in the same way. Such examples can be found in mangrove foci of Guinea, or humid savannahs in Chad, for instance.

The very earliest campaigns based on active case detection and treatment also incorporated interventions against the fly. The mobile teams of Jamot, Richet, and others who controlled HAT in francophone Africa, used the famous “prophylaxis agronomy” strategy to complete the medical activities. This strategy aimed to destroy the natural habitat of tsetse in order to reduce vector-borne transmission of *T. b. gambiense*. Although not acceptable today because of obvious environmental concerns, it is noteworthy that the principle of combining vector control and medical activities to eliminate *T. b. gambiense* was understood a long time ago.

Recent advances in our understanding of the epidemiology of Gambian HAT have strengthened the case for an integrated approach. First, mature *T. b. gambiense* infection rates in tsetse (i.e., when trypanosomes have reached the salivary glands of tsetse and can be transmitted to the next mammalian host) are usually below 1% even in active foci (Jamonneau et al., 2004), and this is also true for *T. b. rhodesiense* (Auti et al., 2012). This means that reduction in tsetse densities, even those not reaching total elimination, will also reduce transmission. Re-analysis of the theoretical models developed by Artzrouni and Gouteux (1996) suggest that transmission can be halted without the elimination of tsetse (Hastings, unpublished data).

Second, there is more and more evidence that some people can live a long time while being infected by *T. b. gambiense* (Bucheton et al., 2011; Jamonneau et al., 2012). This is the case for asymptomatic carriers, but also for some seropositive people who are not detected by the parasitological techniques and who will develop the disease (Bucheton et al., 2011).

These two elements strongly suggest that without vector control to break the transmission cycle, elimination of *T. b. gambiense* cannot be achieved even under the assumption that HAT is an anthroponosis (i.e., transmission does not involve a non-human reservoir host). If non-humans are important reservoir hosts (Funk et al., 2013), then, as with Rhodesian HAT, elimination of Gambian HAT can only be achieved through a combination of medical activities and vector control.

In addition to the technical arguments, tsetse control may make active case detection and treatment more efficient and affordable. Active case detection and treatment rarely covers more than 80% of the community treated. And it is recognized that the people who are not screened are the ones who are the most exposed (farmers, fishermen, hunters, people who work in plantations): Laveissière and Penchenier (2005) estimate that when 75% of the total population is attending a HAT medical survey, only 50% of the cases are detected. As a result, transmission of *T. b. gambiense* will still continue after a medical intervention unless vector control is also carried out. In the absence of a vaccine or prophylactic drugs, vector control offers the only means of protecting people from infection while also reducing transmission of trypanosomes from the residual population of HAT-infected people. We suggest therefore that adding a vector control component will increase the sustainability of medical interventions.

## Is vector control affordable and achievable?

Restricted application of insecticide to cattle has proved cost effective and successful (Torr et al., 2007) and this technique currently provides good control of *rhodesiense* form HAT in Uganda (Welburn et al., 2006) and so has proved affordable and achievable. However, this approach to HAT control only works where cattle densities are high enough, which is not the case for most areas with *gambiense* HAT. Where cattle densities are not high enough, insecticide treated cloth targets or cloth traps are often used and have been used successfully (Lancien, 1991; Laveissière and Penchenier, 2005) and currently tsetse control operations conducted in Gambian HAT foci use this type of technology (Kagbadouno et al., 2011, 2012). In all situations, the vector control phase should be implemented after a first phase of baseline data collection that will help to precisely define the identity, density, and spatial distribution of the targeted tsetse species. Until recently, tsetse control operations have been said to be unaffordable, however recent work has changed this (Lindh et al., 2009; Rayaisse et al., 2010; Esterhuizen et al., 2011). This more affordable means of vector control is discussed below.

**Figure 1:**
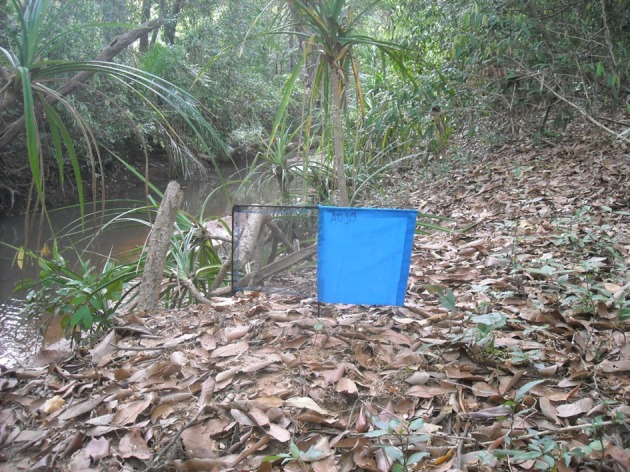
**Picture of a recently developed insecticide impregnated tiny target deployed on the bank of a river in a forest gallery**.

Most gambiense-HAT is transmitted by “Palpalis group” tsetse, more commonly known as riverine tsetse. If we look more closely at the known distribution of HAT cases and the distribution of tsetse flies, we can see that the vast majority of current transmission is being caused by only two tsetse species, *G. fuscipes spp*. and *G. palpalis spp*. We have been concentrating on producing cheaper, target-based control technology for these species with funding from the Bill and Melinda Gates Foundation. A major discovery is that very small targets (see Figure 1) are highly effective for these two species (Rayaisse et al., 2010; Esterhuizen et al., 2011). This completely changes the prospects for use of tsetse control in campaigns against *gambiense* form HAT. Commercial companies can provide long-lasting versions of these insecticide impregnated targets at ~$1 each. Reducing target size will not only reduce material costs but also promises significant savings in operational costs. The large size of standard target designs means deployment is difficult and slow. Shaw et al., 2007, using data from control operations conducted using large targets, estimated that the overall mean rate of deployment was one target per person per day. We believe that the tiny targets now available will increase deployment rates considerably and the savings associated with this increased productivity will be significant. Once deployment rates reach >4 targets per person per day we estimate the costs fall to between US$50 to 100 per km^2^ per year which makes vector control a more feasible proposition for those involved in HAT control.

## Who should do the control?

Tsetse control can be effectively undertaken at a range of levels from regional activities involving several countries to the local village level. A possible scenario could be that control activities should be planned, organized, and implemented at the scale of the focus, by national HAT control programs with personnel from health or vector control structures who already work in the focus. Clearly at the beginning, baseline data collection and the first vector control activities should be organized professionally by a national team who has the expertise of doing it, or who has been trained to have such expertise. Then, most of the activities (target maintenance, deployment, and tsetse densities monitoring) could be progressively transferred to NGOs or local health workers, with some supervision from the national team.

## Conclusion

A change of paradigm has occurred, since it is now recognized that vector control is part of the elimination strategy of gambiense HAT (WHO, 2012), as a complement to medical activities. Integrating medical and vector-based interventions will enable HAT-affected countries to eliminate Gambian HAT.
